# Supplementary material for: Nutritional Status, Dietary Intake and Dietary Diversity of Landfill Waste Pickers
Source: Nutrients. 2022 Mar 10;14(6):1172. doi: 10.3390/nu14061172 (PMC8954570; doi:10.3390/nu14061172)
Supplement: Supplementary file 1 [file nutrients-14-01172-s001.zip › nutrients-1548107-supplementary.pdf]

nutrients MDPI

Supplementary Materials

Nutritional Status, Dietary Intake and Dietary Diversity of Landfill Waste Pickers

Elizabeth C. Swart [1,*], Maria van der Merwe [2], Joy Williams [1,3], Frederick Blaauw [4], Jacoba M. M. Viljoen [5] and Catherina J. Schenck [6]

Table S1. Missing values per landfill site.

Landfill site (n max)	General	Anthropometry	Diet	General & Diet	Anthropometry & Diet	Total
**1 (16)**	1	1	0	1	0	3
**2 (58)**	5	1	0	4	0	10
**3 (38)**	0	0	3	0	1	4
**4 (30)**						
**5 (33)**	1	0	1	0	1	3
**6 (24)**						
**7 (102)**	8	0	18	1	3	30
**8 (49)**	14	1	4	1	9	29
**9 (34)**	2	1	1	2	1	7
**Total (409)**	**31**	**4**	**27**	**9**	**15**	**86**

Table S2. Body mass index of participants.

	**Females**	**Males**	**All**
N			
valid	164	222	386
missing	10	12	23
Mean	26.4162	20.8663	23.2243
Median	25.2871	20.3605	21.5614
SD	6.32838	2.92625	5.42372
Minimum	15.21	13.67	13.67
Maximum	48.01	32.92	48.01
Percentiles			
25	21.3120	19.0538	19.4528
50	25.2871	20.3605	21.5614
75	31.1318	22.3106	25.3141

Table S3. Body mass index classification according to landfill site (N = 386).

**Site**		**Weight status (WHO criteria)**			
		**Underweight (≤18.5)**	**Normal weight (18.5–<24.9)**	**Overweight (25–29.9)**	**Obese (≥30)**
1	n (%)	11 (19.0%)	37 (63.8%)	7 (12.1%)	3 (5.2%)
2	n (%)	1 (5.6%)	15 (83.3%)	2 (11.1%)	0
3	n (%)	4 (10.5%)	17 (44.7%)	9 (23.7%)	8 (21.1%)
4	n (%)	2 (6.7%)	14 (46.7%)	4 (13.3%)	10 (33.3%)
5	n (%)	10 (30.3%)	22 (66.7%)	0	1 (3.0%)
6	n (%)	3 (12.5%)	19 (79.2%)	2 (8.3%)	0
7	n (%)	2 (2.0%)	61 (59.8%)	20 (19.6%)	19 (18.6%)
8	n (%)	12 (24.5%)	36 (73.5%)	0	1 (2.0%)
9	n (%)	2 (5.9%)	15 (44.1%)	10 (29.4%)	7 (20.6%)
Total	n (%)	47 (12.2%)	236 (61.1%)	54 (14.0%)	49 (12.7%)

Table S4. Summary of food consumption per site by food group.

Site	n	1	2	3	4	5	6	7	8	9	Total
**Sample size**		**54**	**18**	**35**	**30**	**32**	**24**	**87**	**47**	**32**	**359**
Cereals	n	53	18	34	30	31	24	85	46	32	353
	%	98.2	100	97.1	100	96.9	100	97.7	97.9	100	98.3
White roots and tubers	n	10	6	11	10	16	6	11	24	7	101
	%	17.9	33.3	31.4	33.3	50	25	12.6	51.1	21.9	28
Vitamin A rich vegetables and tubers	n	1	0	0	0	0	5	0	3	0	9
	%	1.8	-	-	-	-	20.8	-	6.4	-	2.5
Dark green leafy vegetables	n	9	4	5	9	2	3	21	2	11	66
	%	16.1	22.2	14.3	30	6.3	12.5	24.1	4.3	34.4	18.3
Other vegetables	n	5	3	9	5	2	13	4	5	5	51
	%	8.9	16.7	25.7	16.7	6.3	54.2	4.6	10.6	15.6	14.1
Vitamin A rich fruits	n	0	0	0	0	0	0	0	0	0	0
	%	-	-	-	-	-	-	-	-	-	-
Other fruits	n	5	7	3	0	2	0	8	10	3	38
	%	8.9	38.9	8.6	-	6.3	-	9.2	21.3	9.4	10.5
Organ meat	n	1	0	2	0	0	0	9	0	4	16
	%	1.8	-	5.7	-	-	-	10.3	-	12.5	4.4
Flesh meats	n	31	17	26	15	16	22	38	34	22	221
	%	57.4	94.4	74.3	50	50	91.7	43.7	72.3	68.8	61.6
Eggs	n	3	2	2	1	2	0	6	5	1	22
	%	5.4	11.1	5.7	3.3	6.3	-	6.9	10.6	3.1	6.1
Fish and other seafood	n	8	0	4	5	2	2	4	2	2	29
	%	14.3	-	11.4	16.7	6.3	8.3	4.6	4.3	6.3	8
Legumes, nuts and seeds	n	3	1	1	2	3	1	0	6	3	20
	%	5.4	5.6	2.9	6.7	9.4	4.2	-	12.8	9.4	5.5
Milk and milk products	n	24	7	9	9	6	7	5	11	8	86
	%	42.9	38.9	25.7	30	18.8	29.2	5.8	23.4	25	23.8
Oils and fats	n	19	14	25	13	8	17	33	33	16	178
	%	35.2	77.8	71.4	43.3	25	70.8	37.9	70.2	50	49.6
Sweets	n	36	12	25	17	20	19	56	38	26	249
	%	66.7	66.7	71.4	56.7	62.5	79.2	64.4	80.9	81.3	69.4
Spices, condiments and beverages	n	32	8	17	12	20	15	43	28	15	190
	%	59.3	44.4	48.6	40	62.5	62.5	49.4	59.6	46.9	52.9

Table S5. Consumption of foods and beverages that are not part of a healthy eating plan.

Site	n	1	2	3	4	5	6	7	8	9	Total
**Food/Beverage**		**54**	**18**	**35**	**30**	**32**	**24**	**87**	**47**	**32**	**359**
Processed meats	n	6	3	12	9	3	2	6	9	2	52
	%	10.7%	16.7%	34.3%	30%	9.4%	8.3%	6.9%	19.2%	6.3%	14.4%
Alcohol	n	9	2	5	1	3	2	4	0	0	26
	%	16.1%	11.1%	14.3%	3.3%	9.4%	8.3%	4.6%	-	-	7.2%
Sugar-sweetened beverages	n	24	7	19	9	5	9	29	23	13	138
	%	44.4%	38.9%	54.3%	30%	15.6%	37.5%	33.3%	48.9%	40.6%	38.4%
Fruit juice	n	0	0	0	0	0	0	1	0	1	2
	%	-	-	-	-	-	-	1.2%	-	3.1%	0.6%

Table S6. Average individual dietary diversity score per site.

Site	n	Mean	Standard deviation	Minimum	25%	50%	75%	Maximum
**1**	56	2.519	0.771	1.0	2.0	2.0	3.00	3.0
**2**	18	3.167	0.858	2.0	3.0	3.0	4.00	4.0
**3**	35	2.543	0.780	1.0	2.0	2.0	3.00	3.0
**4**	30	2.433	0.774	1.0	2.0	2.0	3.00	3.0
**5**	32	1.969	1.204	0.0	1.0	2.0	2.25	3.0
**6**	24	3.167	0.917	2.0	3.0	3.0	3.25	4.0
**7**	87	2.069	0.789	0.0	2.0	2.0	2.00	3.0
**8**	47	2.575	0.950	1.0	2.0	2.0	3.00	3.0
**9**	32	2.813	0.931	1.0	2.0	3.0	4.00	4.0
